# A Developmental Approach to Predicting Neuronal Connectivity from Small Biological Datasets: A Gradient-Based Neuron Growth Model

**DOI:** 10.1371/journal.pone.0089461

**Published:** 2014-02-21

**Authors:** Roman Borisyuk, Abul Kalam al Azad, Deborah Conte, Alan Roberts, Stephen R. Soffe

**Affiliations:** 1 School of Computing and Mathematics, Plymouth University, Plymouth, United Kingdom; 2 School of Biological Sciences, University of Bristol, Bristol, United Kingdom; 3 Institute of Mathematical Problems in Biology of the Russian Academy of Sciences, Pushchino, Russia; Georgia State University, United States of America

## Abstract

Relating structure and function of neuronal circuits is a challenging problem. It requires demonstrating how dynamical patterns of spiking activity lead to functions like cognitive behaviour and identifying the neurons and connections that lead to appropriate activity of a circuit. We apply a “developmental approach” to define the connectome of a simple nervous system, where connections between neurons are not prescribed but appear as a result of neuron growth. A gradient based mathematical model of two-dimensional axon growth from rows of undifferentiated neurons is derived for the different types of neurons in the brainstem and spinal cord of young tadpoles of the frog *Xenopus*. Model parameters define a two-dimensional CNS growth environment with three gradient cues and the specific responsiveness of the axons of each neuron type to these cues. The model is described by a nonlinear system of three difference equations; it includes a random variable, and takes specific neuron characteristics into account. Anatomical measurements are first used to position cell bodies in rows and define axon origins. Then a generalization procedure allows information on the axons of individual neurons from small anatomical datasets to be used to generate larger artificial datasets. To specify parameters in the axon growth model we use a stochastic optimization procedure, derive a cost function and find the optimal parameters for each type of neuron. Our biologically realistic model of axon growth starts from axon outgrowth from the cell body and generates multiple axons for each different neuron type with statistical properties matching those of real axons. We illustrate how the axon growth model works for neurons with axons which grow to the same and the opposite side of the CNS. We then show how, by adding a simple specification for dendrite morphology, our model “developmental approach” allows us to generate biologically-realistic connectomes.

## Introduction

**The relationship between structure and function of neuronal circuits is a challenging problem in neuroscience and has two related aspects:** 1) How can we identify the neuronal connections which lead to appropriate activity of a circuit? 2) How does that activity, as a dynamical pattern of spiking activity, lead to functions like cognitive behaviour? Experimental neuroscience provides knowledge on mechanisms of spike generation and propagation along the axon, synaptic transmission to other neurons and many other details of neuronal network function. However, in many cases important information about large scale synaptic connectivity (contacts between neurons) is missing. One reason is that the experimental investigation of connections between large numbers of neurons is extremely difficult so there is only limited information on these connections, and their detailed mapping between all individual neurons in all but the smallest networks is absent. A way to address this problem and predict large scale network connectivity on the basis of relatively small amounts of information is through development. Axons typically grow out from the cell body to make synaptic connections with the dendrites of other neurons. If we can define the rules controlling axon growth and their formation of synaptic connections with dendrites by generalizing from a small database of known connections, then we can build a developmental model to generate the axons and connections in a whole network [1]. Rather than trying to estimate connections in a fully formed neuronal network, this paper therefore describes a “developmental” approach to studying anatomical connectivity. At the core of this developmental approach is a new biologically realistic model of axon growth.

**Modelling axon growth is a very active field of research.** The major challenge in this area is to understand, both empirically and theoretically, the mechanisms underlying axon growth, given ever expanding knowledge about the guidance cues and their interactions with the growth cone, the motile and very sensitive structure at the growing axon tip [2]. It is typically assumed in modelling axon growth that axons are guided to their target neurons in the developing nervous system with remarkable precision by sensing molecular cues in the extracellular (growth) environment [3], [4], [5], [6]. The target cells secrete molecular cues and thus create a gradient of increasing concentration which growth cones can sense and follow towards their target [7], [8], [9], [10]. Mathematical models of the growth of axons towards their targets usually rely on sensing of diffusive gradients by filopodia, which are dynamic hair-like protrusions from the growth cones [11]. The molecular cues can be either attractive or repulsive to the growth cone [12], [13]. Mathematical models of axon growth were developed in [14], [15], where axons grow in a two-dimensional plane governed by differential equations for the locations of the growth cones, coupled to diffusion equations that describe the gradient field set by the diffusive attractive and repulsive guidance cues. Such models can generate realistic axons which grow towards their target cells.

**The vertebrate spinal cord offers an example where axons grow to form functional networks as a result of interactions with molecular and physical cues in their environment** [**16**] **,** [**17**] **,** [**18**] **,** [**19**] **.** Evidence suggests that molecular cues, secreted from the dorsal roof plate and ventral floor plate of the cord, are initially responsible for establishing a dorso-ventral series of longitudinal columns of distinct neuron types on each side of spinal cord [20], [21]. Later in development the same morphogen cues may then act as axon guidance cues [22]. Some neuron types have axons growing on only one side of the cord, while others have commissural axons, attracted to the ventral floor plate and then crossing ventrally to the opposite side [23]. After crossing, these commissural axons are transformed and no longer attracted to the ventral floor plate [24], [25], [26]. Instead, they turn to grow longitudinally [27] like their ipsilateral (uncrossed) counterparts, either towards the head or the tail or branching to grow in both directions. In this study, we present a biologically-tractable mathematical model for axon growth and the formation of synaptic connections. The model incorporates the complex responses of the growth cone to gradient fields from its initial emergence from the cell body through different stages of axon growth to produce detailed, biologically-realistic patterns uncrossed and crossing projection.

**Our growth model is of the neurons in the spinal cord and brainstem of newly hatched frog tadpoles opened like a book to make it two-dimensional.** Research here has provided detailed anatomical and functional information on the networks controlling swimming [28]. Electrophysiological recordings from pairs of neurons have revealed synaptic connections [29], [30], [31], [32]. The results led to a proposal that the location or geography of axons and dendrites plays a fundamental role in establishing connectivity [1]. For example, if “geographically” the dendrites of some neurons are located mainly dorsally while the axons of other neurons are located mostly ventrally, then it is unlikely that they will form synapses. It is important to note that in this case, as is widespread for CNS networks, axons grow and make connections along their length rather than seeking specific, distant targets and their overall growth trajectory is therefore critical. Our previous simple mathematical model of axon growth allowed us to generate large networks whose connectivity could be analysed [1], [33], [34] but it was limited in important ways. It only considered axon growth after it had reached a longitudinal orientation and axon guidance was based on fixed values of artificial parameters, in particular a simple “attractor”. It did not model the usually-ventral initial outgrowth of the primary axon from the soma, the orientation to longitudinal growth, branching to form a secondary axon, or the formation of commissural projections. By addressing these limitations, our new model allows more useful biological interpretation.

**Our present aim was to build a gradient-based model for growth of whole neuron morphologies based on biologically-plausible responses to axon guidance cues provided by rostro-caudal (longitudinal), dorsal and ventral morphogen gradients.** When dendrites were allocated to neurons, this developmental approach [1], [35] could be used to assemble complete networks of neurons in the tadpole spinal cord based on limited biological datasets. Fundamental to this approach is generalization from measured data where the number of recorded cases is rather limited. We did this for key model features by generating large sets of values whose probabilistic structures matched those of the limited anatomical datasets. In this way we can model the complete development of the specific morphology and resulting synaptic connections of the seven types of neurons in the tadpole swim network. The process starts with the assignment of soma position, grows an entire primary axon, followed by branching to grow a secondary axon. It then allocates dendrites and allows probabilistic synapse formation between axons and dendrites which come into contact, with a probability based on measurement [1]. The details of this modelling process are specific for each neuron type. In the Discussion we consider the wider utility of our approach.

## Materials and Methods

### Mathematical Formulation of Axon Growth Model

#### Derivation of difference equations of axon growth

The derivation of axon growth difference equations (dynamics in discrete time) follows the work of Krottje and van Ooyen [15]. This approach considers how the tip of a growing axon is guided by gradients representing spatial differences in the concentrations of diffusive or molecular cues. Some terms therefore describe features of the environment in which the axon is growing (these are mainly considered in the Results), while others describe the response of the growing axon to those features. Here we derive a system of three nonlinear difference equations that describe a process of axon growth under the assumption that the two-dimensional growth environment, including the concentrations of molecular cues, remains steady.

We start with a general formulation of the mathematical model, which is used to grow each fragment of an axon and where the parameters of the model are biologically tractable. This model is convenient, flexible and biologically plausible; therefore we hope the model will have wider utility. Any particular application of the model requires adjustment of model parameters according to the specific details of a particular biological system. Here, it has been used to generate different parts of the axon projections of different types of tadpole spinal neuron. We begin from mathematical formulation and in the Results section show specific examples of neuron growth.

**Figure 1:**
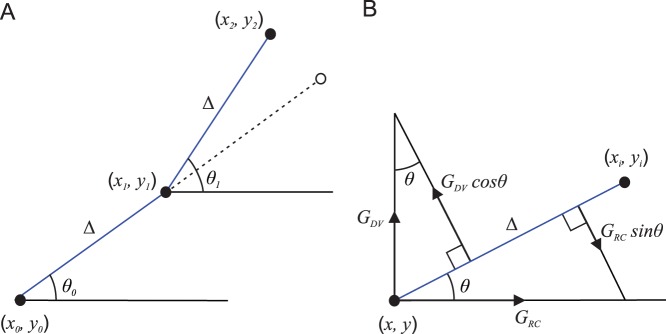
Features of the axon growth process (A) Three consecutive points of a growing axon are shown. Direction of growth during each step (*Δ*) is defined by the growth angle (*θ*). The dashed line shows the trajectory if based only on axon “stiffness” (keeping the same direction) where there is no influence causing it to deviate. (B) Rostro-caudal (*G_RC_*) and dorso-ventral (*G_DV_*) gradients and their projections to the direction of growth between two consecutive points of a growing axon.

The axon grows in discrete steps and this growth process is studied in a two-dimensional representation of the spinal cord with co-ordinates 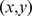, where 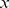 is the rostro-caudal (longitudinal) position along the body and 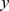 is the dorso-ventral position on one side of the body. These co-ordinates are measured in micrometres. The growth dynamics are also characterised by a growth angle 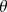. The dynamics of this angle are characterised by “stiffness”, the tendency of the growing axon to grow straight, keeping the same growth angle as was used on the previous growth step, and an “ability to deviate”, which is the tendency of the axon to deviate from a straight path according to the influence of environmental cues (Fig. 1). The addition of a random variable at each step of growth provides an additional degree of freedom for axon growth. In fact, interplay between environmental cues and this random perturbation defines a key feature of the axon growth model. Also, the random variable makes it possible to generate computationally a set of axons with similar statistical properties to real axons [36].

Figure 1 illustrates the model derivation. The “stiffness” is shown in Figure 1A by the dashed line; however, environmental influences change the angle value, and growth from the point with co-ordinates 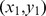 to the point with co-ordinates 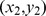 is characterised by angle 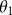 which can be different from the previous angle 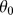 (hence “ability to deviate”). Figure 1B illustrates the influences of two gradients resulting in a change of the angle value: a rostro-caudal gradient 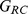 and a dorso-ventral gradient 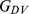. Influences of these gradients on the growth angle are characterised by deviations in a direction perpendicular to the direction of axon growth. Each gradient is therefore projected to a direction perpendicular to the current growth to describe the change of the growth angle. Thus, the model is described by the following difference equations:
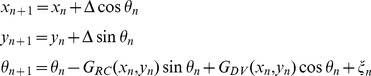
(1)where 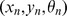 are the current co-ordinates of the axon tip and the growth angle at step 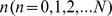; 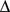 is the axon elongation at each step (usually 1 µm); 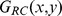 and 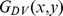 are rostro-caudal and dorso-ventral gradients, whose influence will depend on the current position of the growth cone; 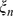 is the value of a random variable acting on the current step of the growth process (here, a uniform random variable in the interval 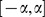). This system of three nonlinear difference equations (1) provides a general mathematical formulation of the model which, although intended for application to tadpole spinal neurons, can be considered as a computational kernel that can easily be adapted to take into account other specific biological features.

The effects of the rostro-caudal and dorso-ventral gradients are actually an interaction between two components: the environmental cue itself and the sensitivity of the axon tip to that cue. The resulting influence depends on the position of the axon tip:
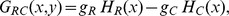


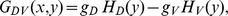
where 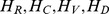 describe the gradient cues while functions 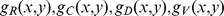 describe the sensitivities of the axon tip to each element of the gradient field.

Each environmental gradient cue is described here by a decaying exponential function:
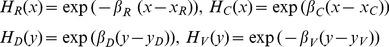
(2)where parameters 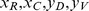 specify the rostral, caudal, dorsal and ventral edges for the four gradient cues (where the each is at its maximum value) and parameters 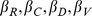 specify their decay rates. Thus, exponential functions with these parameters describe the properties of a common environment in which all the axons grow, and which is identical for the growing axons of all different neuron types. The values of these parameters are therefore chosen to be the same when generating axons of all neurons, independently of their type.

In contrast, the sensitivities of the axon tips to the gradient cues 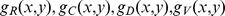 and the random variable 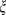, which describes a stochastic component of axon growth, are specific for different neuron types.

The model of axon growth (1) can now be re-written in the following form:
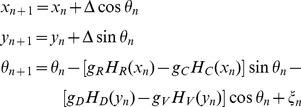
(3)


#### Adjusting the equations of axon growth for the specific case of the tadpole spinal cord

Having described the derivation of the difference equation for basic axon growth, we next describe how the equations (2–3) are modified and extended to apply them to the specific biological example of the tadpole spinal cord.

The first modification is to assume that either the rostro-caudal gradient 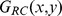 or the dorso-ventral gradient 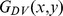 can be represented by a single component. For the tadpole, we have simplified the first of these by assuming that axon growth in a longitudinal direction is only influenced by a rostral gradient. Axon growth ascending (towards the head) or descending, (away from the head) can then be controlled by either an attractive or repulsive sensitivity of the axon tip to this gradient. A second modification is to give the rostral gradient cue a slope of zero so that 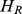 is a constant and the cue acts as a polarity; i.e. it signals rostral direction but not position. Biologically, this could represent an electrical field rather than a chemical cue [37] or the behaviour of an axon tip that used sensitivity to concentration difference (e.g. between sides of the growth cone) rather than absolute concentration [38] to detect direction or polarity. This rostro-caudal polarity cue is uniform and therefore no longer depends on the rostro-caudal position of the axon tip. The model of axon growth is then described by the following equations:
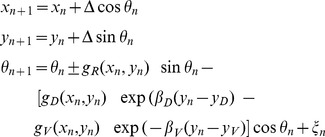
(4)here, the sign “+” corresponds to growth in the ascending direction and the sign “-” corresponds to descending growth. Values of the environmental parameters used here for modelling the tadpole are: 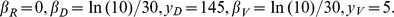 These values are motivated by biological plausibility and the form of the growth field representing the tadpole hindbrain and spinal cord. In fact, there is little experimental evidence on the decay rate of gradients [39]. Suggestions range from shallow gradients [40] to relatively steep gradients [41]. The values we have chosen for the tadpole are based on multiple simulations using a variety of decay rates. We have found that precise values are not critical; a range of relatively fast decaying gradients will work well for the model provided that sensitivities to gradients and the random factor are adjusted appropriately for the chosen environment.

For modelling the primary axons of tadpole neurons, we consider three consecutive stages of axon growth. The first is the **outgrowth** stage. This is controlled by a fixed set of parameter values. For uncrossed axons, this stage is very short, but for crossing axons growth continues ventrally until axons have travelled through the floor plate and emerged on the opposite side. The second stage is an **orientation** stage in which typically-ventral growth turns to become longitudinal, either ascending or descending depending on neuron type. Parameter values are not fixed during this stage but change smoothly between two different sets depending of the axon length (see below). The third stage is the **main** stage of longitudinal growth. Like the outgrowth stage, this is controlled by a fixed set of parameter values. Secondary axons, which branch from the primary axon and run in the opposite longitudinal direction, have only a single **main** stage of growth that is controlled by a fixed set of parameter values. (These stages, as well as branching, are considered further in the Results.).

During the **orientation** stage of growth, the sensitivities of a growing axon tip change along the axon growth path and therefore depend on the co-ordinates of the axon tip. The functions 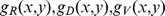 (which are constant during the **outgrowth** and **main** stages of growth) now describe how axon tip sensitivities change according the axon length:
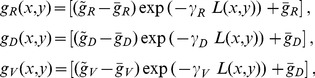
(5)here: 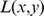 is the length of the growing axon from the start of the orientation stage to the current point; parameters 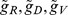 are the sensitivity parameters of rostral, dorsal and ventral gradient cues respectively at the start of the orientation stage; 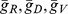 are sensitivity parameters for rostral, dorsal and ventral gradients respectively for the subsequent, main stage of axon growth, and therefore the end of the orientation stage; 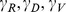 are decay rates for rostral, dorsal and ventral sensitivity functions respectively and describe the smooth transition between sensitivity parameter values during the orientation stage.

These equations (5) reflect the biological situation where axon behavior changes significantly during a stage of axon growth and where a transition between two set of sensitivity parameters is needed to model this change appropriately. In the tadpole case, the sensitivity parameters differ in magnitude but, more generally, they could also be of different signs indicating a change between attraction and repulsion for the particular environmental cue.

In an analysis of a simplified version of system (4), assuming that sensitivities to gradients are constant (Supporting Information S3 “Mathematical analysis of difference equations of axon growth”), the dynamics can be compared with the behaviour of axons described by our previous model (see equations (1) in paper [33]). This previous model is almost linear and its dynamics are characterised by an asymptotic tendency to some dorso-ventral attractor. Although the new model is highly non-linear, it is proven that in this simplified case the growing axon approaches some dorso-ventral position, where two gradients are balanced. However, the use of a non-linear equation for growth angle means that the dynamical behaviour of the new model is more flexible and complex than the previous almost-linear model [42]. It is worth emphasising that the sensitivities in model (4) are not constant but depend on the position of axon tip; in fact, these functions depend on the current length of a growing axon. Also, these functions can be changed after crossing the ventral floorplate when the sign of sensitivities is reversed.

### Generalization from Biological Data

To start iterations of the axon growth model (4) we have to specify the initial values of several variables before running the simulation: the co-ordinates of the starting point 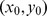 (at the soma if growing a primary axon or at a branch point if growing a secondary axon); the outgrowth angle 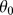, which specifies the initial direction of growth; and the axon length. These values are specific for each neuron type and we use available anatomical measurements to define them using a computational procedure called Generalization from biological data. The importance of the generalization procedure is that it allows approximation to a measured distribution; therefore generated data have random probability distributions which are close to the probability distributions of the original, limited, measured biological data. This procedure helps ensure that the modelling of axon growth is biologically realistic.

#### Computational procedures to generalize information from limited biological data

We describe here two generalization methods that were used to construct random distributions, based on available biological measurements, from which sensible values could be selected.

##### One-dimensional generalization from cumulative distributions

In this case we generalize from a one dimensional sample (of size *k*). First, the cumulative distribution function is constructed from this sample. Second, a piece-wise linear approximation of the cumulative distribution function is considered. This approximation is a continuous, monotonic function. Therefore, to generate the generalised value we use the following algorithm: generate a uniformly distributed random value *w* in the interval [0, 1] and use this value as the vertical co-ordinate of the piece-wise linear approximation; a projection to the abscissa (horizontal axis) gives the generated value.

**Figure 2:**
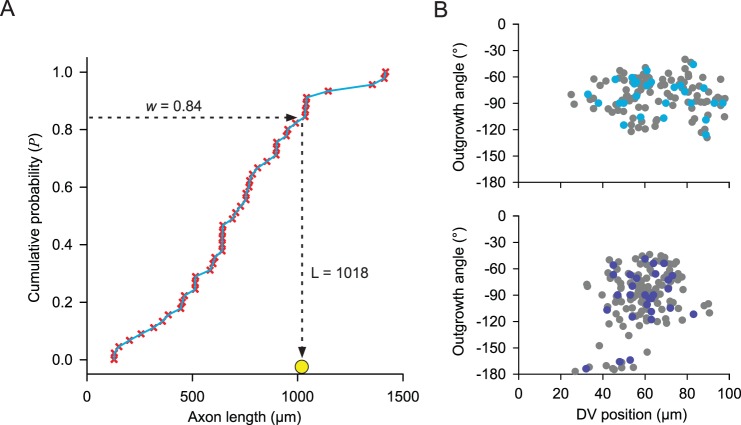
Generalization of values from limited biological datasets. (A) Piece-wise linear approximation of the cumulative distribution function for cIN axon length. Red stars are points on the cumulative distribution function whose horizontal co-ordinates relate to biological measurements (*n = *46). The blue line shows the piece-wise-linear approximation of the cumulative distribution. The yellow dot shows the generated axon length corresponding to random value *w*. (B) Two-dimensional generalization of dorso-ventral axon start point and axon initial outgrowth angle for two examples of tadpole spinal neurons (cIN upper, aIN lower). Coloured symbols are measured values; grey symbols are generated values. (Algorithm parameter values: 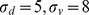 and 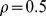; see SI for details.).

An example of one dimensional generalization is illustrated in Figure 2A to specify axon length for tadpole ‘cIN’ neurons [28]. In terms of our model (4), axon length defines the total number of iterations (axon length is the number of iterations times the prolongation due to one iteration; here 1 µm). The sample size for cIN axon length is *k = *46 and lengths lie in the range 110 µm to 1,450 µm (see the longitudinal axis in Fig. 2A). The cumulative distribution function is constructed and the piece-wise linear approximation is shown by the blue line. Horizontal co-ordinates of red stars correspond to the sample. To generate the axon length we randomly select a probability value from interval [0, 1] (*w* = 0.84) and project it to the horizontal axis. In Fig. 2A an arrow points to the generated axon length (L = 1018 µm), shown by the yellow circle.

##### Two-dimensional generalization

A different approach can be used where the biological data are represented by ordered pairs of measurements. For example, values for the initial angle of axon outgrowth may be related to the dorso-ventral co-ordinate of the start of the axon. In this case, a two-dimensional generalization procedure is needed. A sample of pairs of DV position and initial outgrowth angle is shown in Fig. 2B by coloured dots for cIN and aIN neurons (upper and lower plots respectively). Grey dots show generalised values which are obtained using the two dimensional normal distributions around sample dots. For details of the algorithm, see Supporting Information S1 “Two-dimensional generalization procedure”. This method is also used for assigning dendrites (see below).

### Stochastic Optimization of Axon Growth Parameters

#### Stochastic optimization of model parameters for axon growth

The previous section described how to define initial values for variables of system (4) and values for some of the growth model parameters, which are specific for the axon growth of each neuron type. Parameter values for the ‘outgrowth’ and ‘orientation’ stages of primary axon growth (described above) were chosen visually in such a way as to suitably describe a specific shape of axon during these initial stages of growth. For the orientation stage, this involved specifying the sensitivity parameters 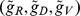, which describe how the growing axon tip responds to the gradient cues in the growth environment at the start of this stage, and the exponential decay rates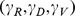, which describe the transition to their ‘main’ axon growth stage values (Eq. 5).

A different approach was used to define the parameters needed to model the ‘main’ stages of primary and secondary axon growth. The main stage of axon growth is characterised by its own sensitivity parameters 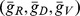. To define values for these three parameters as well as the fourth parameter 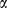, which provides an interval of variation of the uniform random variable 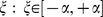 (see equation (4)), an optimization procedure is needed. The optimization procedure used should provide the best values for the four axon growth parameters 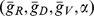, corresponding to the smallest value of a designated cost function (see below). This cost function is designed in such a way to match the model-generated axons to the real, measured axon samples for a particular type of neuron.

Optimization of the four axon growth parameters for a selected neuron type starts with generation of a set of modelled axons to be compared with measurements of real axons from the same neuron type. Here, measured, real axons and modelled axons were located on a two-dimensional rectangular plan inspired by the biological reality (explained in the Results section). Parameter values for start position, initial growth angle and axon length were specified using the generalization procedures described above. Some starting values were required for the four growth parameters. Initial starting guesses for these values were then changed at each iteration step of the optimization procedure. Where the iterations converge, the result of the final iteration provides the best parameter values corresponding to the smallest value of the cost function (i.e. the cost function value closest to zero since the cost function could be positive or zero).

#### Design of the cost function

**Figure 3:**
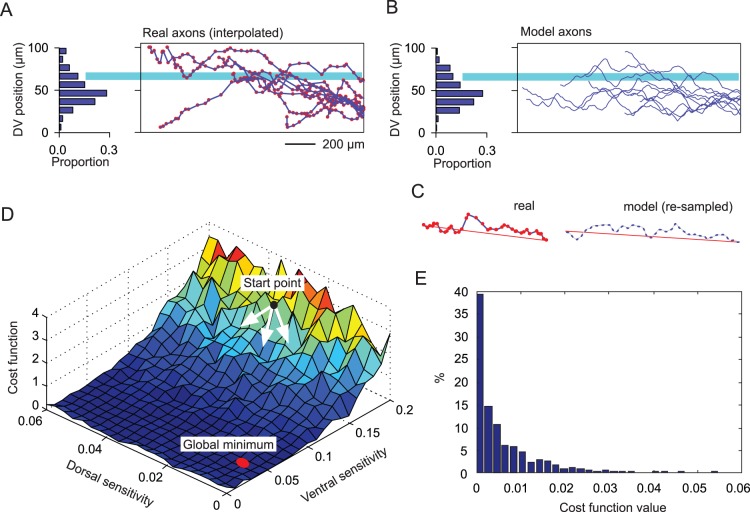
Cost function components and optimization of growth parameters for tadpole aIN neurons. (A) Ten axon trajectories. Histogram (left) showing the dorso-ventral distribution of interpolated points along the length of a set of real axons (right: viewed laterally as in Fig. 4C; red symbols indicated intermittently measured points with all axons starting at the right). The proportion of points accumulated at each dorso-ventral level (e.g. cyan bar) is shown in the appropriate 10 µm bin. (B) Like A, but for a set of model axons. (C) Tortuosity in single axons. Red lines indicate the direct (chord) length; red symbols indicate measured points on the path of a real axon (left); paths of model axons (right, blue) were re-sampled at 10 µm intervals. (D) The random component in the cost function needed for optimization produces an uneven surface (illustrated for two dimensions: dorsal and ventral sensitivity). White arrows indicate multiple slopes from the start point of a search for a minimum cost function value (Global minimum). (E) Histogram of a sample containing 1000 repetitive calculations of the cost function for a single set of axon growth parameter values (All examples in A–E are for tadpole aINs).

The cost function used to measure similarity between the generated and real axons of tadpole spinal neurons comprised components based on two simple features that describe the main trajectories of the axons well: the dorso-ventral distribution of points along their length and their tortuosity (wiggliness). The dorso-ventral distribution was found simply by projecting points along the length of the axon to the vertical axis and counting them in 10 µm bins (Fig. 3A,B). For model axons (Fig. 3B), all points were generated at 1 µm step intervals. Measurements of real axons (Fig. 3A) were made intermittently along their length, typically at mean intervals of ∼10 µm. To make these measurements comparable to those from model axons, a simple linear interpolation procedure was first used to link the measured co-ordinates with others at 1 µm intervals. Similarity was estimated using normalised least squares, following the traditional, statistical chi-square approach (see Supporting Information S2 “Defining the cost function for stochastic optimization” for further details). The tortuosity (*T*) of each axon is the ratio of the total path length (arc) to the straight line distance between start and end points (chord) (Fig. 3C; see Supporting Information S2 “Defining the cost function for stochastic optimization” for details of calculation). To make tortuosity values for real and model axons comparable, model axon co-ordinates were first re-sampled at 10 µm intervals along their length, similar to the spacing between measurements of real axons. A squared difference between average tortuosity values of real and generated axons was then used as a measure.

The two terms of the cost function, the similarity between dorso-ventral projection distributions and the similarity of axon tortuosities, have very different scales. To balance them, we therefore used a weighting coefficient 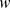 to make these terms of the same order. Thus, the final expression for the cost function is:
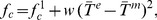
(6)where 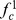 describes the similarity between dorso-ventral axon distributions (see Supporting Information S2 “Defining the cost function for stochastic optimization” for details of its calculation) and 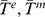 are the average tortuosities of real and modelled axons respectively; the weighting coefficient 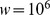.

#### Optimization procedure

Optimization of the axon growth model parameters 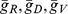 and 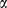 requires minimizing the cost function. However, the axon growth model (Equations 4) includes a random variable 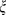 so the cost function behaves irregularly like a random variable, and the surface of this function in four-dimensional space is not smooth but uneven (Fig. 3D). Also because the cost function behaves as a random variable, each successive calculation of the cost function under the same fixed parameter values will generate a slightly different value of the cost function. For these reasons, a stochastic optimization technique is required. Therefore, to minimize the cost function and find a set of optimal parameter values for 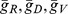 and 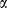, the ‘pattern search’ algorithm was applied (for mathematical details see [43]). This very efficient algorithm belongs to a class of direct search methods where solving an optimization problem does not require any information about the gradient of the cost-function. The optimization procedure combines a random search on a mesh of variable size with finding a descending direction and calculation of the cost function along this direction. A “patternsearch” routine from the Global Optimization Toolbox of MATLAB was used.

The strength of this method can be illustrated by considering an optimal set of axon growth parameter values obtained for the primary axons of one specific tadpole spinal neuron type (aIN, see below). For this set of values 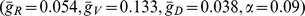 we repeated the calculation of the cost function one thousand times, resulting in a sample of different cost function values, all calculated for the same fixed parameter values. The statistical characteristics of this sample of cost functions were: minimum: 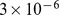; maximum: 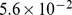; mean: 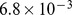; and standard deviation: 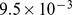. It is clear from a histogram of the sample (Fig. 3E) that about 40% of cost-function values are located in the first bin with its centre at 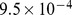. Thus, simple statistical analysis shows that optimal parameter values provide small values of the cost function and means that the quality of optimization, as in the case of these aIN neuron primary axons, is good. For detailed evaluation of optimization quality we consider the optimal parameter values and generate 100 primary axons which are used to compare their tortuosities and dorso-ventral distribution “histograms” with those of experimentally measured axons. First, we apply a two sample t-test to compare the differences between tortuosities of modeled and real axons. For each generated axon we calculate the tortuosity and do the same for each measured axon. The statistical test shows that the difference between the means of the two sets of tortuosities (modeled and real axons) are not significant (p-value>0.05). Second, we compare “histograms” of modeled and measured axons. Strictly speaking, the standard statistical tests are not applicable for estimating the similarity between “histograms” of dorso-ventral axon distributions. The reason is that the data in the sample are not independent because they are close successive points on the same axon. Nevertheless, we apply a two-sample chi-square test to compare histograms for generated and real axons which confirms the similarity of “histograms” (p-values>0.05).

### Generating Connections from Grown Axons

“Growth” of a network of interconnected neurons (connectome) using the axon growth model followed a series of stages: distribution of neuron somata; assignment of dendrites; growth of axons; and formation of synapses where axons and dendrites meet. The following briefly describes each of these stages for generating a tadpole connectome (further details are given in the Results section below). The various parameter values needed are summarised in Table S1 “Connectome generation parameters”.

**Figure 4:**
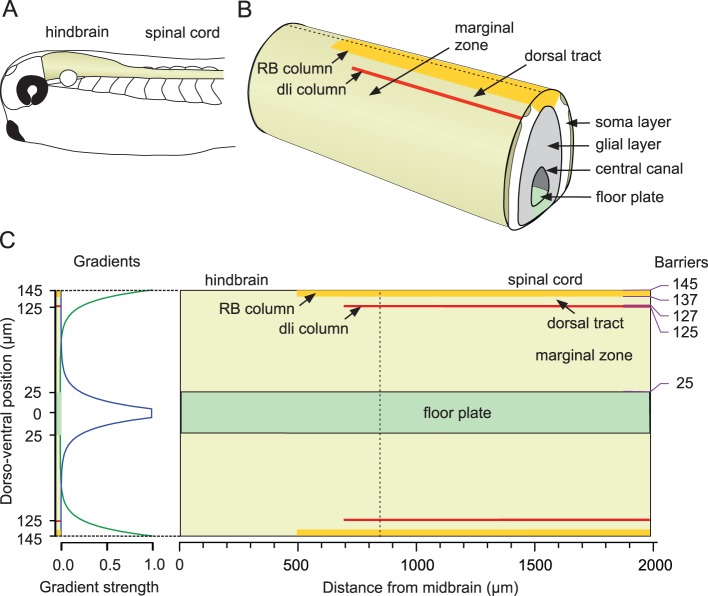
The two-dimensional environment for axon growth. (A) Side view of the head end of the tadpole showing hindbrain and spinal cord (buff). (B) Diagram of a section of the CNS to show the main parts including the central canal surrounded by a glial cell layer and the ventral floor plate, surrounded in turn by the layer of neuronal somata. Lying outside the soma layer are the marginal zones, in which most axons grow, and the dorsal tracts containing sensory neuron axons, separated from the marginal zone by a barrier formed by the dli column, a column of dorsolaterally-situated sensory pathway somata (red line), and bounded dorsally by the column of RB sensory neuron soma (yellow line). (C) The CNS opened like a book along dorsal midline (dotted line in B). Graphs on the left show the gradients originating at the dorsal edge (G_D,_ green) and near the midline of the ventral floor-plate (G_V_, blue). There is also a longitudinal polarity (G_R_, not illustrated). Lines on the right (purple) indicate the dorso-ventral positions of a series of barriers to axon growth (see text for further details).

Neuron somata were placed rostro-caudally within the growth environment (see Fig. 4) based on data of their real numbers and distributions [1]. Different neuron types were assigned consecutively, with all individuals at minimum longitudinal separations of 1.5 µm. The algorithm used (see Supporting Information S4 “Soma distribution” for details) contains a random component, so the distributions were different each time they were generated, but their statistical properties were the same. The dorso-ventral position was assigned using the two-dimensional generalization procedure, which also generated the initial, outgrowth angle of the axon (see above). The co-ordinates given by the rostro-caudal and dorso-ventral position of each soma define the origin of the axon for that neuron.

Dendrites were then placed at the rostro-caudal position of each soma. The detailed shape of each dendrite was not modelled here. Instead, dendrites were represented by a vertical bar between dorsal and ventral extremes whose values were obtained from real data using the two-dimensional generalization procedure described above. This simple representation is sufficient to allow realistic synapse formation. In the tadpole spinal cord, all synapses are thought to be made from axons onto dendrites, rather than being axo-somatic or axo-axonal.

**Figure 5:**
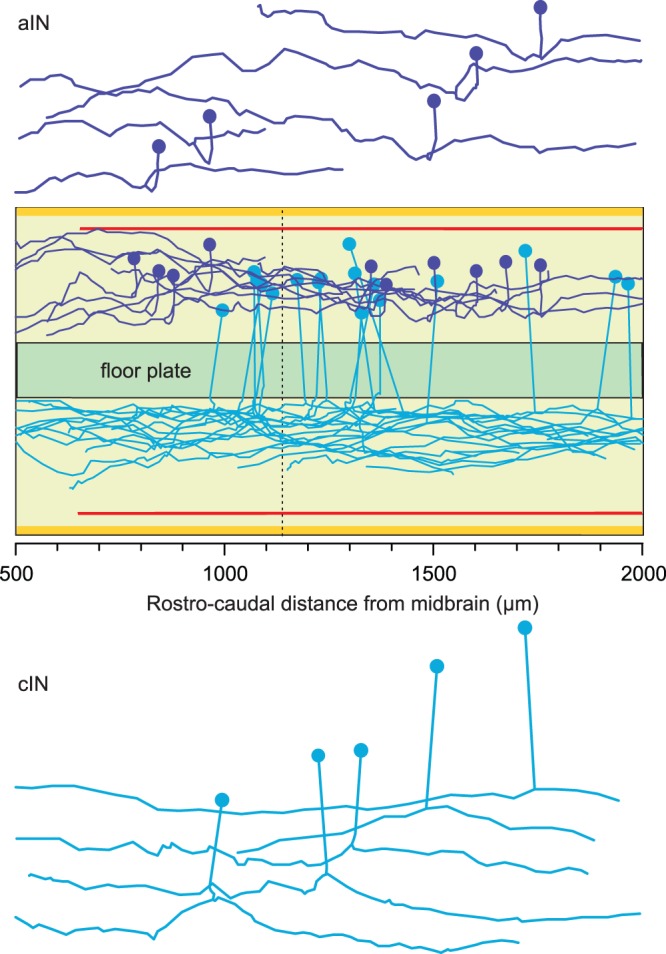
Measured axon projections of two of the tadpole neuron types: aINs (dark blue) with uncrossed, primary ascending axons and secondary descending axons; and cINs (light blue) with crossing, primary ascending axons and secondary descending axons. In each case, examples are shown *in situ*, within the growth environment and also individually to illustrate their basic morphology.

Next, the growth model was used to generate an axon starting at each of the neuron somata. For many neurons, based on biological measurements and as described above, growth of a primary axon was followed by growth of a secondary axon from a branch point on the primary axon (see Fig. 5).

Lastly, synaptic connections were allowed to be made where the axon of one neuron met the dendrite of another neuron. On the basis of electrophysiological evidence from paired recordings, the probability that such a meeting would actually produce a synaptic contact was set at 0.46 for most pairs but 0.63 for sensory pathway connections [1].

## Results

### Using Biological Details to Specify the Environment for Axon Growth in the Spinal Cord of the Young *Xenopus* Tadpole

The original motivation behind the computational model described here was its specific application to neuron growth in a simple vertebrate system, the spinal cord of the 48-hour post-fertilization hatchling *Xenopus* tadpole. Here we introduce biological details of the *Xenopus* tadpole system and describe application of the general mathematical model of axon growth (see previous section) to the tadpole spinal cord. The adjusted model takes into account specific details of axon growth for different spinal cord neuron types. Because the different populations of spinal cord neurons, and particularly many of their ascending axons, typically extend in to the hindbrain, the model considers growth in both hindbrain and spinal cord.

#### Young frog tadpole CNS anatomy

The 48-hour old hatchling *Xenopus* tadpole is 5 mm long and the eyes are not yet functioning but the brain and spinal cord contain differentiated neurons. The spinal cord is a simple tube about 100 µm in diameter with a central canal formed by glial cells and the ventral floor plate ([44]; Fig. 4). On each side lies a layer of neurons loosely organized into longitudinal columns. The neurons project axons in the longitudinal direction, either directly or after first growing ventrally across the floor plate to the other side and then turning or branching longitudinally. Axons lie in the marginal zone on the outside of the spinal cord and can grow more than 1,000 µm (20% of the body length), and wander dorsally and ventrally as they grow.

The tadpole CNS tapers towards the tail. For modelling purposes, we therefore consider axon growth only in a 2,000 µm length of the CNS, including the hindbrain and the first 1,150 µm of the spinal cord, where the degree of tapering is relatively small. For modelling, this approximately-cylindrical region of the CNS is transformed into two dimensions by ‘cutting’ it along its dorsal midline (where no axons cross), opening it out flat and viewing it from the outer surface. In plan view, the axon growth area then becomes a pair of rectangles, one for each side of the cord, separated by a further rectangle representing the floor plate (Fig. 4C). The justification for this transformation into a two-dimensional plan-view is that the outer layer of each side of the spinal cord in which the main axon growth occurs (the ‘marginal zone’) is approximately 100 µm in dorso-ventral extent and (for the part considered here) 2,000 µm long, but it is only ∼10 µm in thickness. Therefore, in ignoring the thickness of the growth area and considering it in just two dimensions, there is very little compromise anatomically. However, there is a significant simplification computationally. Generation of axons within the growth area is then controlled by gradient fields and constrained by a series of anatomical barriers (Fig. 4C). These elements are considered in turn.

#### Spinal gradient fields

It is known that in the developing vertebrate spinal cord, neurons arise from progenitor cells in the neural tube [45]. The hypothesis that forms a basis for our model is that guidance molecules along the spinal cord set up gradient fields which steer axons into appropriate locations and thus ensure the formation of proper connections [46]. In the part of the hatchling *Xenopus* tadpole CNS that we are considering here, three possible sources of guidance molecules that could attract or repel axons are: the dorsal roof plate, the ventral floor plate and the hindbrain (Fig. 4C) [17], [5], [19]). Because the proposed guidance molecules are diffusive, the gradients following their changes in concentration are considered to be exponential in form. The second important element of axon growth is the sensitivity of the growing axons to the gradient fields. Since the axons of tadpole spinal neurons make synaptic connections with the dendrites of other neurons *en passant* along their length, the path followed by the growing axon, rather than a specific destination, is probably a key to the developing pattern of connectivity between neurons.

In the model, there are three line sources for the guidance cues which influence axon growth in the rectangular CNS growth area: one 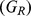 starting at the midbrain-hindbrain border (the origin on the longitudinal axis); one 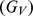 starting 5 µm from the midline of the ventral floor plate, which is at 0 along the dorso-ventral axis; and the last 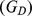 starting at the dorsal edge, which is at 145 µm from the ventral midline. The assigned values for the slopes of the gradient cues are 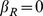, 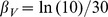 and 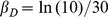 (see above for comments on selection of these values). In the specific application of the model to the tadpole CNS, the formulation of a slope value in the form of 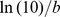 is convenient as the value 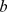 for a gradient cue is the distance over which the gradient strength decreases to 10%. The longitudinal signal is given a constant value. Biologically, this implies a polarity along the rostro-caudal axis causing turning of an axon in the longitudinal direction. The gradient slopes are kept fixed for all neuron types so that the axonal growth cones of all neurons are subjected to same environmental cues. On the other hand, growth cone sensitivities to the gradients differ, so axons of different spinal neurons respond differently to the same gradient fields.

#### Barriers to axon growth

In addition to gradient sources, the model includes five barriers running longitudinally on each side of the CNS, most representing tightly packed rows of neuron somata which axons do not cross (purple lines, Fig. 4C). Axons approaching these barriers are deflected. In the model, the axons are turned longitudinally upon contact. The way the axons are restricted at the barriers and deflected along them reflects what is observed biologically. The most ventral barrier is at the outer margin of the floor plate, which is the ventral edge of the marginal zone (25 µm from the ventral midline). There are two barriers at the dorsal edge of the marginal zone and the ventral edge of the dorsal tract (125 and 127 µm from the ventral midline). These barriers are formed by a column of sensory pathway neuron somata (dli column, Fig. 4C) and extend forward to 700 µm from the midbrain. A further barrier at the top of the dorsal tract (137 µm from the ventral midline) is imposed by the column of Rohon-Beard (RB) sensory neuron cell bodies which extends forward to 500 µm from the midbrain. A final barrier (145 µm from the ventral midline) imposes a dorsal limit to growth for the most rostral part of the growth area.

### Simulation Results for Axon Growth in Two Different Tadpole Neuron Types

The focus of this section is to illustrate the results of the optimization procedure as applied to the growth model and to demonstrate that the growth model can successfully generate realistic axon projection patterns of two tadpole spinal neuron types using these optimized parameters. First, we introduce the basic morphology of two tadpole spinal neuron types with crossing and non-crossing axons. Then we describe the sequence of stages in the generation of whole axons using the growth model, followed by examples of axon growth in the two spinal neuron types.

#### Spinal neurons and their morphology

As in all vertebrates, newly formed neurons in the tadpole spinal cord lie in a broadly dorsal to ventral sequence: sensory neurons, sensory interneurons, premotor interneurons, motoneurons. There are remarkably few types of spinal neuron, possibly less than ten [47]. Seven neuron types control swimming, the principal behavioural response of the young tadpole [28]. The projection patterns of the growing axons of all spinal neurons fall broadly into two distinct groups: those with uncrossed (ipsilateral) axons, like aINs, and those with crossing (commissural) axons like cINs (Fig. 5). For aINs, a primary axon projects longitudinally towards the head and, at a variable distance from the soma, a secondary axon branches from the first axon and projects towards the tail. For cINs, the primary axon crosses the ventral floor plate to the opposite side and then turns to project longitudinally towards the head; at which point a secondary axon branches from the first and projects towards the tail.

#### Stages of axon growth simulation

**Figure 6:**
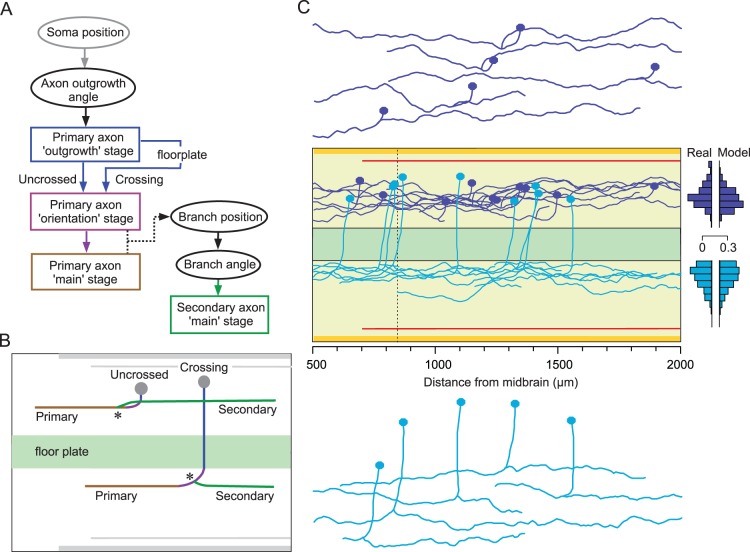
Stages of axon growth and model axon projections. (A) Flowchart summarising the sequence of stages in modelling axon growth for neurons with crossed or uncrossed axons. Rectangles denote axon growth stages; ovals denote values obtained using generalization procedures. Note that a secondary axon can branch from the ‘orientation’ or ‘main’ region of a primary axon. (B) Illustration of the main stages of axon growth described in A. In these examples, both primary axons are ascending. Asterisks indicate branch points. (C) Axon projections generated by the growth model for uncrossed aINs (dark blue) and crossing cINs (light blue). Ten examples of each type are shown *in situ* with some of each type separated to show their individual morphology. Compare to real examples in Figure 5. Bar charts compare the proportions of the main growth for the primary axon projections in real and model axons in 10 µm dorso-ventral bins (projections sampled every 1 µm).

In practice, we found that a realistic axon trajectory usually has a complex shape and to approximate the biological axon, the model needed a sequence of stages (Fig. 6A flowchart). For non-crossing neurons, primary axon growth started with a brief ‘outgrowth’ stage followed by an ‘orientation’ stage in which typically-ventral growth of the axon from the soma altered to become longitudinal (Fig. 6B purple). Orientation was then followed by the ‘main’ longitudinal growth stage (Fig. 6B brown). The transition from the orientation stage to the main stage of primary axon growth was set arbitrarily for all neurons at 100 µm from the axon origin at the soma, measured longitudinally. For crossing neurons, primary axon growth started with an ‘outgrowth’ stage in which axons grew ventrally until they reached and penetrated the floor-plate at ∼90° to cross to the opposite side (Fig. 6B, blue). There was then, again, an orientation stage in which transverse growth became longitudinal, leading to a main growth stage. Transition from the orientation stage to main growth in crossing axons occurred at the point of axon emergence from the floorplate. Model parameters for primary axons were obtained using the optimization procedure (see previous section) only for the main stage of growth, from 100 µm. Axons were generated during outgrowth and orientation stages using the same growth model parameters as for the main growth stage; however, the axon trajectories were quite predictable and setting suitable values for these parameters did not require optimization. Secondary axon growth involved only a single ‘main’ stage from a branch point on the primary axon or from the soma (Fig. 6B, green). Parameters for secondary axons were optimised from their start point at the branch; the position of the branch point and the branch angle were obtained using the one-dimensional generalization procedure.

#### Generating a population of neurons with realistic non-crossing axons

The population of aINs provides an example of neurons with uncrossed axons. It extends from the caudal hindbrain along the spinal cord. All aINs have an uncrossed, ascending primary axon which usually gives rise to a descending secondary axon from a branch point close to the soma [47]. [48]. In the simulation (Fig. 6C dark blue) ten axons from aINs are shown. The parameter values for the short ‘outgrowth’ stage and the start of the ‘orientation’ phase of primary axon growth were: 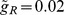, 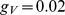 and 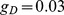, and the optimized values for the main growth were 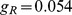, 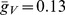 and 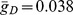. During the orientation stage, the starting values made smooth transitions to their final values, changing exponentially (Eq. 5) with decay rates 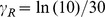,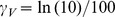 and 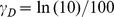 respectively. Stochasticity was given by 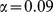. The secondary axons were adequately generated using the same parameters as for the main, primary axon growth.

#### Generating a population of neurons with realistic crossing axons

Like aINs, the population of cINs extends from the caudal hindbrain down the length of the spinal cord. The primary cIN axons are initially directed ventrally, like those of aINs, but continue to grow ventrally, enter the ventral floor plate and cross to the opposite side (Fig. 6C light blue). During modelling, the trajectory of this outgrowth stage was directed by the initial growth angle, and by weak rostral and ventral attractions: 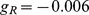, 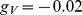 and 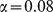. On leaving the floor plate on the other side, cIN primary axons then turn to project longitudinally. This change to a longitudinal path was controlled during the orientation stage by a smooth transition from a starting set of parameter values: 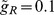, 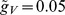 and 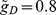 to a final set of values optimized for the main stage of ascending axon growth: 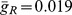, 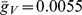 and 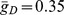. This transition during the orientation stage was governed by the exponential functions described by equations (5) with the decay rates 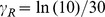,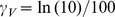 and 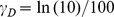 respectively. Stochasticity was given by 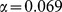. Most cINs have a descending, secondary axon that arises as a branch on the primary axon once it has crossed ventrally and emerged from the floor plate. The position of the branch and the initial axon growth angle for the secondary axon were obtained by one-dimensional generalization. Optimized secondary axon growth parameter values were: 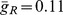, 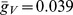, 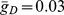 and 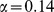.

Simulation of the axons of these two examples of spinal neurons shows that the growth model based on axon guidance by a gradient field can generate biologically realistic morphologies of both crossing and non-crossing neurons in the tadpole spinal cord. The similarity between the dorso-ventral distributions of real and model axons is shown by the bar charts in Figure 6C.

#### Simulation software

All simulations in this section were performed using software called SC2D (“spinal cord in 2 dimensions”), which provides a framework for axon growth of different types of neurons in a two dimensional environment. This code was written for MATLAB and runs on a standard PC computer. The code is available on request.

#### Longitudinal axon growth and the importance of the random variable

**Figure 7:**
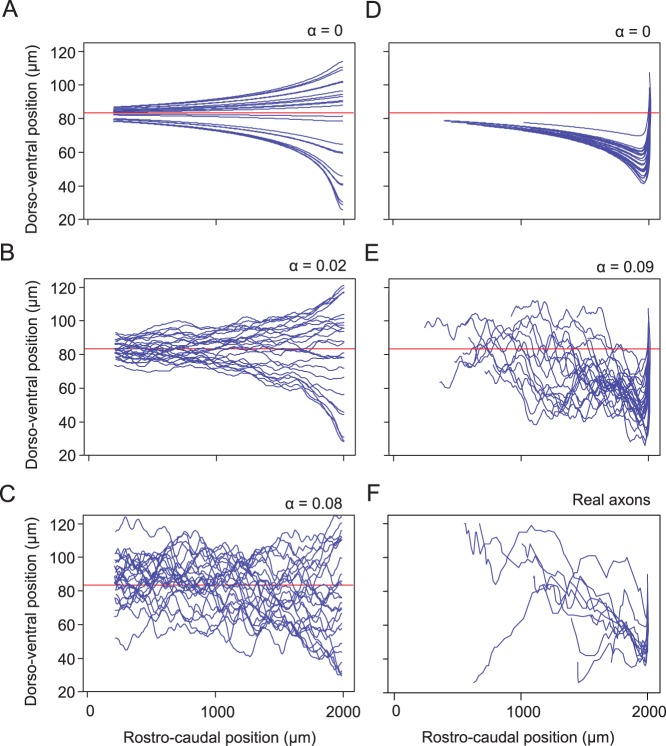
The influence of the random variable and initial angle on ascending axon growth. Groups of 25 ascending axons, grown using aIN parameters. Note: All axons start at 2000 µm rostro-caudally and from a range of dorso-ventral positions. The fixed point of stability for aINs is indicated (red line shows 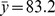). (A–C) Axon starts are randomly distributed dorso-ventrally. As the random variable is increased (values of α indicated), trajectories become more variable: for 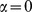, axon trajectories approach 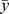; for 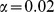 and 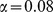, trajectories increasingly deviate from 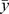. (D)(E) Axons have lengths, dorso-ventral start positions and initial angles distributed according to generalized aIN values. With 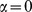, no axons reach 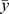 within the length of the axon. With 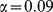, the value optimized for aINs, the tendency of growth towards 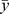 is much less obvious, but the axon trajectories are much more realistic. (F) Ascending axons of real aINs, aligned rostro-caudally to match the model axons.

Mathematical analysis of the difference equations of axon growth shows that axon growth monotonically along the longitudinal co-ordinates (either in the ascending or descending direction) tends asymptotically to some particular dorso-ventral position, designated 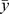 A particular value of 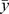 depends on the sensitivity parameters 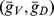 (see Supporting Information S3 “Mathematical analysis of difference equations of axon growth” for details of the analysis and the derivation of 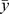). This latter tendency is heavily influenced by the random variable 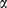, which controls the shape of the generated axon (Fig. 7A–C). Optimal aIN parameters (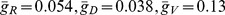, which give a value of 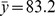) were used to generate 50 axons with their initial positions uniformly distributed dorso-ventrally between 25–145 µm. The initial longitudinal position was fixed at 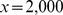 µm and axons were grown in the ascending direction (outgrowth angle 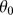 = 180°). Figure 7A shows how axons look if they are generated without the random variable (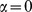). It is clear that axons tend towards 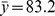, as calculated above and indicated by the red line. In figure 7B, 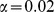 and the influence of the random variable is rather weak. The tendency towards 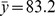 is still visible (red horizontal line) despite random perturbations in axon growth trajectory. In figure 7C, 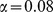 (this value is close to the optimal value 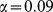) and exerts a larger effect; the tendency towards 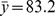 is largely obscured by the random perturbations. With much higher random perturbations (e.g. 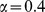), very large random deviations are produced where the axon trajectory varies wildly and may loop in a way that is not observed biologically (not illustrated).

A group of aIN axons growing with no random variable are also shown in figure 7D (with the same parameter values and initial longitudinal position as above). However, in this case, the axons were made more biologically realistic with axon lengths and outgrowth angles selected randomly from measured aIN values using the generalization procedure (see section above). The initial ventral growth means that axons still tend towards 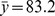 but may not approach it closely before they terminate. Introducing the random variable, optimized for aINs (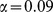; Fig. 7E), produces biologically realistic axon trajectories (compare with real trajectories for the same neuron type, Fig. 7F), where the tendency of growth towards 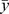 is much less clear. In reality, this will allow a dorso-ventrally distributed set of axon trajectories for each neuron type despite the underlying tendency to align to a specific dorso-ventral level.

### Generation of an Example Connectome

**Figure 8:**
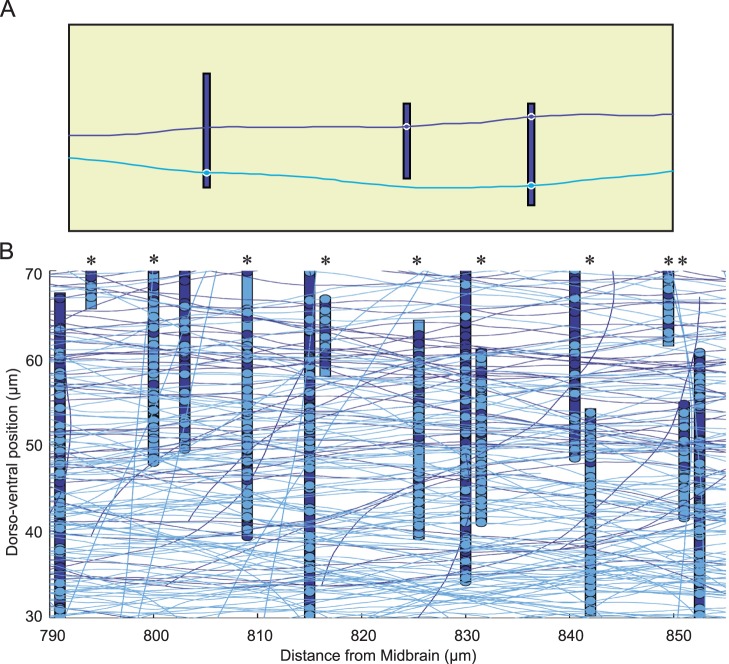
Connectome generation. A. Diagram with two longitudinally-running axons passing the dendrites (vertical bars) of three aIN neurons. Synapses (circles) can form (with probability = 0.46) where an axon meets a dendrite. B. Part of the growth field showing a region of a partial connectome formed by populations of just two neuron types (aIN, dark blue and cIN, light blue). Asterisks indicate dendrites of cINs.

The primary aim of the work described here was to build a computational model of axon growth but this model forms the core of a novel developmental approach to assembling large networks of interconnected neurons (connectomes). The first two stages of this process were the assignment of soma positions and dendritic extents within the growth environment. The significance of each soma position is simply that its co-ordinates are the start point for growth of the axon, and also the rostro-caudal position of its dendritic “field”, designated simply by a dorsal and ventral extent (Fig. 8A) based on two-dimensional generalization. Following axon growth using the appropriate parameters for each neuron type, synaptic connections could form where the axon of one neuron met the dendrite of another neuron. The probability that a meeting would actually produce a contact was set at 0.46 for most pairs but 0.63 for sensory pathway connections, based on electrophysiological evidence from paired recordings [1], so not all axon-dendrite meetings produced a synapse (Fig. 8A). Figure 8B illustrates a portion of a partial connectome using just two neurons types (aIN and cIN). The trajectories of most axons are primarily longitudinal. Other directions are mainly axons in their orientation stage after leaving the soma or emerging from the floor plate (cIN). The total number of aIN neurons on one side is 68 and the total number of cIN neurons on the same side is 192. Growing axons of these 260 neurons produce 17,725 synapses on one side of the spinal cord. Thus, figure 8B clearly shows that even a small portion of the connectome looks very complex.

## Discussion

### A New Approach to Establishing Complete Connectivity

In this paper we present and discuss the derivation and operation of a model for generating axons in a two-dimensional environment, under the control of a set of gradient cues. The specific goal was to develop a model system that could be used as part of a developmental approach to re-constructing all the neuronal connections in the brain and spinal cord network that controls the locomotor behaviour in a simple vertebrate, the young *Xenopus* tadpole. Even in this simple animal, it would be impossible to establish the complete connectome except by serial EM reconstruction. The crux of the developmental approach was to start with real, though hard to obtain and therefore limited, anatomical data and use these to generate entire axon distributions for realistically large numbers of each type of neuron. This aim could be achieved by generating axon populations whose statistical characteristics matched those of the smaller data sets of real, measured axons for each neuron type. The broader aim in producing this new axon growth model was to provide a tool for generating the axons of any neurons growing in such an environment as a step towards predicting large scale CNS connections. A key feature of this model is that control of axon growth is based on a biologically realistic mechanism: the sensitivity of the growth cone at the tip of the growing axon to gradient cues representing the kinds of diffusible chemical gradients that have been proposed in the nervous system [49], [17]. In this way, the model adds biological reality to an earlier model of axon growth applied to the tadpole spinal cord [1], [33]. Although it was possible with this earlier phenomenological model to fit experimental evidence, the model was formulated in terms of a very simple two-dimensional system of difference equations with parameters which did not correspond to biological reality. In contrast, the current gradient-based model is biologically plausible and its parameters can be interpreted in biological terms.

In the new gradient based model that we present here, we try to keep a balance between important biological details (such as gradient cues to guide the axon growth) and model simplification (such as our coarse grain approach which does not include detailed consideration at the level of growth cone filopodia and molecular mechanisms). Recent work has uncovered many of the molecules which are involved in the process of axon guidance (chemotaxis) however, the nature of any gradients and the mechanisms underlying chemotaxis are still unclear [5], [19], [38], [50]. For a recent review on theoretical modelling of neural development including models of axon growth, see [51]. Methods are starting to be developed for visualizing morphogen gradients [10], but the shapes of those in the tadpole are still unknown. Importantly, our new model includes specific expressions for individual gradients, which can be modified to incorporate future experimentally-determined descriptions of the real gradients.

### Outline of Features of Model

The new gradient model of axon growth that has been developed has been applied to generate biologically realistic sets of axons for different types of tadpole spinal neuron. Although the axon growth model comprises three nonlinear difference equations, a key part of the model is a nonlinear difference equation for the growth angle. This equation also includes projections of three gradients (one rostro-caudal and two dorso-ventral) to the current growth direction.

The model includes two sets of parameters: those which describe a common gradient environment where all axons grow; and a set of sensitivity parameters which are specific for each particular type of neuron and for each part (primary or secondary) and each stage (outgrowth, orientation and main) of the growing axon. Values for some of these specific parameters are obtained from measured data using a generalization procedure, but four of them have been defined using an optimization procedure to fit the model in the best way to real, biological measurements. A chosen cost function, used as the basis for the optimization procedure, includes a random variable and therefore, this function is not smooth and a special algorithm of stochastic optimization had to be used here.

### Generation of Axons with Biologically Realistic Features

This model is relatively simple, and includes only four adjustable parameters (three sensitivities and the range of the random variable). However, it was still possible to obtain a good fit of the model to biological measurements for each type of neuron, including the two examples illustrated here.

The gradient model of axon growth includes a key computational part which is a universal algorithm for axon growth. However, to satisfy the multiple requirements and limitations imposed by different biological realities (e.g. limitations of axon length, physical barriers which impede axon growth in particular locations, the distribution of branching points along the primary axons etc), the model of axon growth has to be combined with additional algorithms to generate biologically realistic axons. For example, it is very important to start each simulation of axon growth from an initial angle in a specific range, representing the initial outgrowth from the soma. To define this range, a generalization procedure was developed for random selection of the initial angles from a distribution which coincides with the distribution of measured initial angles. The model of axon growth is used sequentially with different stages of axon generation for axons which grow on one side and also crossing neurons which grow their axons from one side of the body to the other.

An important feature of the model as applied to the tadpole spinal cord is that axons grow until they reach a particular length rather than a particular target. Early in development, the axons make synapses “en passant” along their length rather than only once they have reached a target. The trajectory itself is therefore important in terms of the dendrites that may be encountered; the final destination may not be. We generalized from a sample of observed axon lengths in the model; in reality we do not know what determines this parameter.

A mathematical study of a simplified version of the model without a random component shows that there is a specific dorso-ventral co-ordinate which attracts a growing axon. However, the random component of axon growth masks the influence of this attractive dorso-ventral position and enables the generation of axons which are similar to the real axons. It is interesting to note that the variance of the random variable needs to be carefully chosen by the optimization procedure.

### Other Models of Axon Growth

Several recent models have considered the details of chemotaxis at the molecular level. For example, in the model of Mortimer et al., [52] each receptor measures the number of unbound - to - bound transitions and this information is important for optimal chemotaxis performance. This study also presented some important conclusions on the nature of noise, which can be used for a better description of the random component of axon growth. Our model includes a uniformly distributed random variable in a suitably small, specified range. The uniform distribution was selected after several trials of other distributions to check the possibility of better fitting of the model to real, biological measurements. Other work has described how the Bayesian approach can be used to model the way axons detect gradients and how this guides axon growth [53], [54]. This probabilistic approach is very powerful and provides optimal chemotaxis even in the case of long axons. Mortimer et al., [52] also compared two different approaches to axon growth: 1) a short step elongation along the same direction plus deviation according to the gradient field; 2) modulation of growth rate with long enough steps along the same direction. It was shown that the first mechanism dominates in steep gradients and the second one is more effective in shallow gradients. In our modelling we are closer to the second approach: a small fixed step size of 1 µm along the same direction and re-calculation of a new direction at every step of the computational algorithm. The selected step size is small enough to allow a good axon flexibility but big enough for effective computational procedure.

Another interesting mathematical model [55] aimed to explain a regulatory mechanism where an axon is attracted or repelled by molecular gradients. It was shown theoretically and confirmed experimentally that the ratio of calcium to cAMP is a trigger changing attraction to repulsion and vice versa. Our model of axon growth also is able to switch from attraction to repulsion. In our modelling of commissural neurons we follow some remarkable experimental findings suggesting that the initial part of the axon is guided by attraction to the ventral gradient cue on one side of body but after crossing to the opposite side this cue become repulsive [24], [25], [26], [56].

### Applying the Model to Other Systems

The model of axon growth and, more broadly, the “developmental approach” to predicting interneuronal connections seems to be general enough for application in other neuronal systems. Although the axon growth model works in two dimensional space, appropriate for the tadpole spinal cord, where axon growth is restricted to a shallow outer layer of the cord, it is easy to expand the model and generalization algorithms for the three dimensional case by adding additional angles describing the growth direction in three dimensional space. Thus, in many cases where generalization from a small amount of real, biological data is needed, an expanded axon growth model and developmental approach can be used as a tool in establishing connectivity between neurons.

It would be interesting to compare our SC2D software for anatomical modelling of growing neuron components (soma, dendrite, growing axon branches) in a two dimensional environment with the simulation tool CX3D [57] for modelling the 3D developmental processes in the neocortex. Combining fundamental ideas and approaches from different simulation tools may result in progress toward a better biologically realistic tool for developmental modelling. Indeed, elements of our previous model [58] have already been used with an extension of the CX3D software to model the growth of axons in cells of the pheochromocytoma cell line 12 (PC12) [59]. Because our new model, while still remaining relatively simple, is more directly rooted in biological processes, through use of gradient-based axon guidance, we expect it to form a more attractive basis for network modelling in other systems.

### Using the Axon Growth Model to Generate a Connectome

Our long-term aim in building neuron growth models is to use them to generate a complete, biologically realistic connection architecture or “connectome” for the network in the tadpole brainstem and spinal cord which controls swimming [33]. The procedure we used here, with the new biologically plausible axon growth model at its core, started with the distribution of ∼2000 neuron somata along the length of a 2-D opened-out CNS and from this generated a map of ∼200,000 synapses. The result of our novel developmental approach is a biologically realistic map of the connections in the spinal cord and brainstem network controlling tadpole swimming, reconstructed on the basis of generalization of data from a limited number of biological measurements. Evaluation of this connectome has required mapping it onto a functional, conductance-based neuronal network model where we can test whether the connections generated by the growth model are able to produce swimming activity in response to “sensory” stimuli. Details of connectome properties and the functional swimming model are described separately [60].

## Supporting Information

Table S1 **Connectome generation parameters.**
(PDF)

Supporting Information S1 **Two-dimensional generalization procedure.** Generalization from biological data.
(PDF)

Supporting Information S2 **Defining the cost function for stochastic optimization.** Similarity between the dorso-ventral distributions of real and model axon co-ordinates; Similarity between the tortuosity of real and generated axons.
(PDF)

Supporting Information S3 **Mathematical analysis of difference equations of axon growth.** The longitudinal growth of axons. Reduction of the axon growth model to a one-dimensional map. Reduction of the axon growth model to a two dimensional map. Application of this analysis to a specific example of model axon growth.
(PDF)

Supporting Information S4 **Soma distribution.** Method for generating a rostro-caudal distribution of soma positions.
(PDF)
