# Supplementary material for: A Developmental Approach to Predicting Neuronal Connectivity from Small Biological Datasets: A Gradient-Based Neuron Growth Model
Source: PLoS One. 2014 Feb 21;9(2):e89461. doi: 10.1371/journal.pone.0089461 (PMC3931784; doi:10.1371/journal.pone.0089461)
Supplement: Table S1 — Connectome generation parameters. (PDF) [file pone.0089461.s001.pdf]

Table S1 Connectome generation parameters

Summary of parameters used for connectome generation together with the methods used to define them (see main text).

Parameter description	Parameter notation	Method to prescribe a value
Environment		
Gradient slopes in dorsal and ventral directions:<br>Gradient slope in rostral direction:<br>Dorsal and ventral boundaries:	β_D, β_V<br>β_R<br>$y_D,\ y_V$	Guidance from literature, computational trials and adjustments
Soma		
Rostro-caudal and dorso-ventral coordinates in μm (the same for current position of the axon tip):	$x,\ y$	Distribution algorithm, 2D generalization
Primary axon		
Current growth angle and initial (outgrowth) angle (radian)	$\theta_n,\ \theta_0$	Iterations of difference equations, 2D generalization
Length from initial point to a current position of the axon tip (μm) :	$L(x,\ y)$	1D generalization
Initial 'orientation' stage sensitivities of rostral, dorsal and ventral gradients respectively:	$\tilde{g}_R,\ \tilde{g}_D,\ \tilde{g}_V$	Visual matching
'Main' stage sensitivities of rostral, dorsal and ventral gradients respectively:	$\bar{g}_R,\ \bar{g}_D,\ \bar{g}_V$	Stochastic optimization
Transitional decay rates for rostral, dorsal and ventral sensitivity functions respectively (smooth transition from initial to regular sensitivity values):	$\gamma_R, \gamma_D,\ \gamma_V$	Visual matching
Variability range for the random factor :	α	Stochastic optimization
Secondary axon		
Current growth angle and initial (outgrowth) angle (radian) :	$\theta_n,\ \theta_0$	Iterations of difference equations, 2D generalization
Length from branching point to current position of the axon tip in μm:	$L(x,\ y)$	1D generalization
'Main' stage sensitivities of rostral, dorsal and ventral gradients respectively:	$\bar{g}_R,\ \bar{g}_D,\ \bar{g}_V$	Stochastic optimization
Variability range for the random factor :	α	Stochastic optimization
Dendrites		
Dorsal and ventral extent respectively (μm) :	(v, w)	2D generalization
