# Supplementary material for: A Developmental Approach to Predicting Neuronal Connectivity from Small Biological Datasets: A Gradient-Based Neuron Growth Model
Source: PLoS One. 2014 Feb 21;9(2):e89461. doi: 10.1371/journal.pone.0089461 (PMC3931784; doi:10.1371/journal.pone.0089461)
Supplement: Supporting Information S1 — Two-dimensional generalization procedure. Generalization from biological data. (PDF) [file pone.0089461.s002.pdf]

Supporting Information S1. Two-dimensional generalization procedure.

This procedure can be used to obtain a random sample of pairs of anatomical values where there may be a relationship between the pairs of real biological measurements (e.g. dorso-ventral soma position and axon initial outgrowth angle, or dendrite dorsal and ventral extents). The anatomical data consist of two values (v, w) . To generalize a set of *N* measurements we represent these pairs of measured coordinates $((v_k, w_k),\ k = 1,...N;)$ on a two dimensional plane and generate a 2-D normal distribution. The algorithm has two steps:

1) randomly and uniformly select one experimental measurement (v_k, w_k);

2) generate a value of for 2-D normally-distributed random variable with the mean (v_k, w_k) and some prescribed values for the standard deviations σ_v, σ_w and the correlation coefficient $\rho > 0$.

Main text figure 2B shows this method applied to paired dorso-ventral soma positions and axon initial outgrowth angles for two tadpole neuron types (aIN and cIN). Figure part A shows an example of dendrite generation for tadpole cIN neurons. Blue circles correspond to measured pairs of the most dorsal and the most ventral coordinates of cIN dendrites (*N* = 34). Grey circles show generated pairs using the two-dimensional generalization procedure with parameters $\sigma_d = 15, \sigma_v = 15$ and $\rho = 0.8$, *N* = 204. A positive correlation between the two sets of measurements provides the possibility to generate data allocated inside an ellipse with a long axis appropriately oriented and this ensures that the distributions of the lengths of measured and generated dendrites are similar; Figure part B shows distributions of length for measured and generated dendrites.

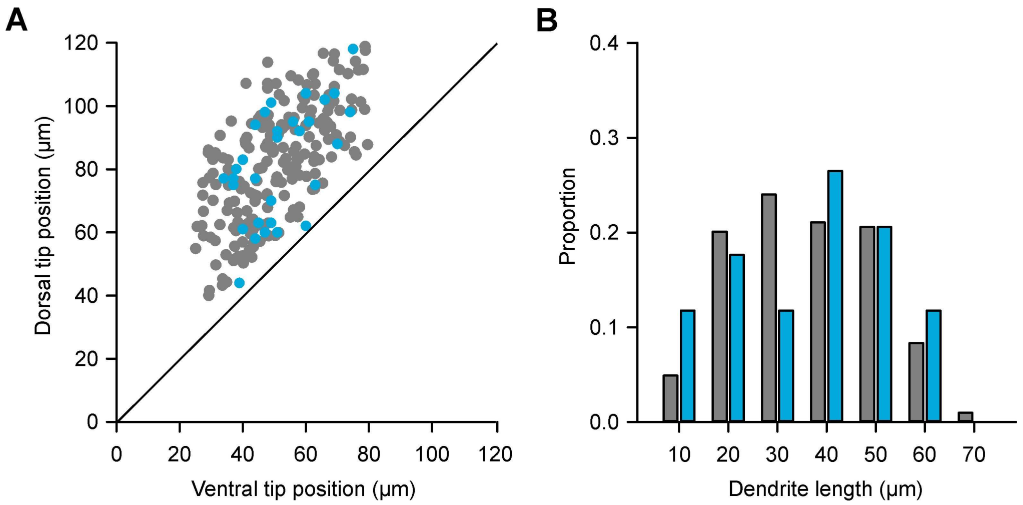

Figure. Using 2-D generalization to assign dendrite extents. A. A set of N = 34 real, measured cIN dendrite coordinates (blue circles). Grey circles are the generated coordinates of 204 cIN dendrites using the following parameter values for the generalization algorithm: $\sigma_d = 15$, $\sigma_v = 15$ and $\rho = 0.8$. B. The length distributions of the measured (blue) and generated (grey) dendrites.
