# Supplementary material for: A Developmental Approach to Predicting Neuronal Connectivity from Small Biological Datasets: A Gradient-Based Neuron Growth Model
Source: PLoS One. 2014 Feb 21;9(2):e89461. doi: 10.1371/journal.pone.0089461 (PMC3931784; doi:10.1371/journal.pone.0089461)
Supplement: Supporting Information S2 — Defining the cost function for stochastic optimization. Similarity between the dorso-ventral distributions of real and model axon co-ordinates; Similarity between the tortuosity of real and generated axons. (PDF) [file pone.0089461.s003.pdf]

Supporting Information S2. Defining the cost function for stochastic optimization

Similarity between the dorso-ventral distributions of real and model axon co-ordinates. Assume (x_i, y_i) denote the rostro-caudal and dorso-ventral coordinates of all generated axon points of all axons with $i = 0,1,2,...,n_m$; where n_m is the total number of generated axon's coordinates (the subscript *m* here means "model"). For the tadpole spinal neurons, the (y_i) coordinates are placed in appropriate bins of a histogram covering the dorso-ventral extent of the growth field on one side; the size of each "histogram" bin is 10 µm and the bins are measured from the lowest ventral coordinate (25 µm; the ventral edge of the marginal zone) till the highest dorsal coordinate (145 µm; the dorsal edge of the cord). Thus, there are 12 bins. The normalised frequencies of model axons for each bin are $p_j^m = \frac{k_j}{\sum_{j=1,12} k_j}$ where k_j is the number of projections in *j*th bin, $j = 0,1,2,...,12$; and the superscript *m* here means "model". The same procedure (projection to the dorso-ventral axis; calculation of binned distributions; and the same 10 µm bin widths) is used for all points of real, measured axons and the resulting frequencies of real, measured axons is denoted by p_j^e , $j = 0,1,2,...,12$, here the superscript *e* means "experimentally recorded biological axons".

To estimate the similarities of real and model axon "histograms", we use the normalised least squares and the normalization follows from the traditional statistical chi-square approach for comparing the two random distributions (histograms): $f_c^1 = \sum_{j=1}^{12} \frac{(p_j^e - p_j^m)^2}{p_j^e n_e + p_j^m n_m}$,

here, p_j^e, p_j^m are frequencies in the *j*th bin, and n_e, n_m are the total number of axon coordinates which have been used to calculate the real and modelled histograms respectively.

Similarity between the tortuosity of real and generated axons. *Real axon trajectories are* circuitous rather than direct and results from multiple model simulations suggest tortuosity as an appropriate measure. Tortuosity (T) is the ratio of the total path length to the straight line distance between start and end points of an axon. For real measured axons, the tortuosity is calculated using measured (uninterpolated) co-ordinates; for modelled axons, the coordinates are re-sampled at 10 µm intervals along the axon trajectory (similar to the spacing between measurements of real axons). A squared difference between average tortuosity of real and generated axons is then used as a measure.

The tortuosity of each axon is calculated using the following formula:

$$T = \frac{\sum_{i=1}^{k} \sqrt{(x_i - x_{i-1})^2 + (y_i - y_{i-1})^2}}{\sqrt{(x_k - x_0)^2 + (y_k - y_0)^2}}$$

where (x_i, y_i) with $i = 0,1,2,..., k$ are coordinates of axon and k is the number of coordinates.
