# Supplementary material for: A Developmental Approach to Predicting Neuronal Connectivity from Small Biological Datasets: A Gradient-Based Neuron Growth Model
Source: PLoS One. 2014 Feb 21;9(2):e89461. doi: 10.1371/journal.pone.0089461 (PMC3931784; doi:10.1371/journal.pone.0089461)
Supplement: Supporting Information S3 — Mathematical analysis of difference equations of axon growth. The longitudinal growth of axons. Reduction of the axon growth model to a one-dimensional map. Reduction of the axon growth model to a two dimensional map. Application of this analysis to a specific example of model axon growth. (PDF) [file pone.0089461.s004.pdf]

Supporting Information S3. Mathematical analysis of difference equations of axon growth

The monotonic growth of axons along the longitudinal direction

In some particular cases the dynamical behaviour of solutions of system (main text Eq. 4) can be studied analytically to highlight certain important features of growing axons. In particular, it will provide a useful comparison between the generation of biologically realistic axons and the behaviour of the underlying difference equations. This Section provides a qualitative analysis for two simplified versions of the axon growth model.

Reduction of the axon growth model to a one-dimensional map. Let us assume that the dorso-ventral gradient and the random variable are absent and the rostro-caudal gradient is constant as well. With these assumptions the axon growth model (main text Eq. 4) is simplified to the following equations:

$$x_{n+1} = x_n + \Delta \cos \theta_n$$
$$y_{n+1} = y_n + \Delta \sin \theta_n$$
$$\theta_{n+1} = \theta_n - \bar{g}_R \sin \theta_n$$

where $\bar{g}_R$ is a positive constant.

The third equation, for the growth angle, is independent of the two other equations. Thus, we study the dynamics of the following one-dimensional difference equation (map):

$$\theta_{n+1} = \theta_n - \bar{g}_R \sin \theta_n.$$

There are two fixed points for this map: $\theta_1^* = 0;\ \theta_2^* = \pi$. To find stability of these fixed points we calculate the derivative of the right hand side of the map:

$$f'(\theta) = 1 - \bar{g}_R \cos(\theta).$$

The fixed point $\theta_1^* = 0$ is stable under the condition $0 < \bar{g}_R < 2$ and the fixed point $\theta_1^* = \pi$ is unstable for any positive value of parameter $\bar{g}_R$. Thus, the growing axon tends to grow monotonically along the longitudinal axis if both the dorso-ventral gradient and the random perturbation are absent. The critical parameter value $\bar{g}_R = 2$ corresponds to the flip bifurcation and for $\bar{g}_R > 2$ the stable 2-cycle appears. It means that the axon growth is in the oscillating regime.

Reduction of axon growth model to a two dimensional map. Next, let us assume that all sensitivities to gradients are constant. In this case the last two equations of the model (main text

Eq. 3) depend on y and θ only. Thus, the model (main text Eq. 3) can be reduced to the following two-dimensional map:

$$y_{n+1} = y_n + \Delta \sin\theta_n$$
$$\theta_{n+1} = \theta_n - \bar{g}_R \sin\theta_n - [\bar{g}_D \exp(\beta_D (y_n - y_D)) - \bar{g}_V \exp(-\beta_V (y_n - y_V))]\cos\theta_n + \xi_n \quad .$$

Also, we assume that the random variable is not present ($\xi = 0$). There are two fixed points in this two dimensional map $(\bar{y},0)$ and $(\bar{y},\pi)$. Here $\bar{y}$ is an intersection of graphs of the dorsal $[\bar{g}_D \exp(\beta_D (y_n - y_D))]$ and ventral $[\bar{g}_V \exp(-\beta_V (y_n - y_V))$ gradients Figure shows these graphs of two exponentials and their intersection $\bar{y}$.

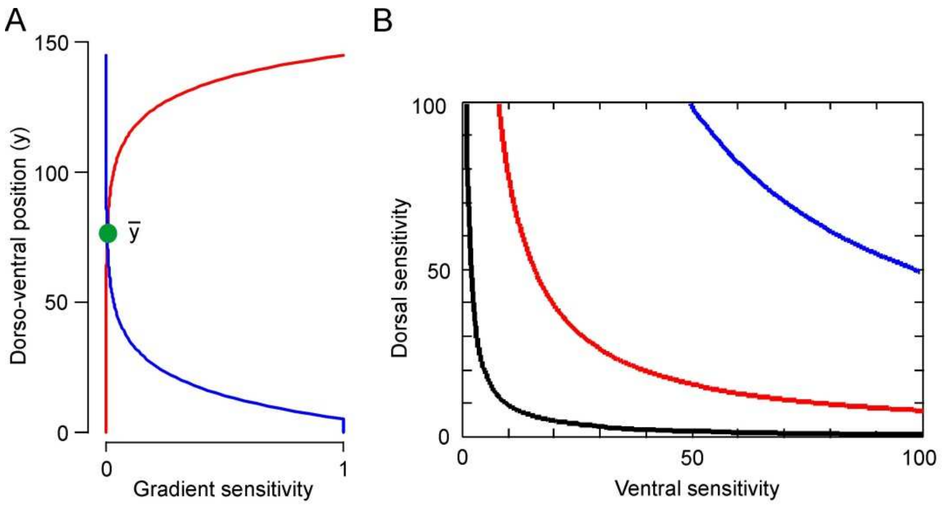

Figure. Longitudinal axon growth. A. Profiles of the dorsal (red) and ventral (blue) gradients with their intersection $\bar{y}$ shown by the green dot. B. Critical boundaries corresponding to different polarities are shown by curves of different colours. For each curve the bottom left corner relates to the stable fixed point $(\bar{y},0)$.

To find the analytical expression for the value of $\bar{y}$ a system for the fixed points is considered (we neglect the random variable and assume that step length $\Delta = 1$):

$$y = y + \sin\theta$$
$$\theta = \theta - \bar{g}_R \sin\theta - [\bar{g}_D \exp(\beta_D (y - y_D)) - \bar{g}_V \exp(-\beta_V (y - y_V))]\cos\theta \tag{S1}$$

It follows from the first equation of system (S1) that either $\theta = 0$ or $\theta = \pi$ and from the second equation the value of $\bar{y}$ is:

$$\bar{y} = \frac{\beta_D y_D + \beta_V y_V + \ln(\bar{g}_V / \bar{g}_D)}{\beta_V + \beta_D} \tag{S2}$$

Stability of these fixed points $(\bar{y}, 0)$ and $(\bar{y}, \pi)$ follows from a consideration of the linearization matrix:

$$J = \begin{pmatrix} 1 & \cos\theta \\ -[\bar{g}_D \beta_D e^{\beta_D (y-y_D)} + \bar{g}_V \beta_V e^{-\beta_V (y-y_V)}]\cos\theta & 1 - \bar{g}_R \cos\theta + [\bar{g}_D e^{\beta_D (y-y_D)} - \bar{g}_V \beta_V e^{-\beta_V (y-y_V)}]\sin\theta \end{pmatrix}$$

For this matrix the trace and the determinant are:

$$Tr = 2 - \bar{g}_R \cos\theta + [\bar{g}_D e^{\beta_D (y-y_D)} - \bar{g}_V \beta_V e^{-\beta_V (y-y_V)}]\sin\theta, \ ,$$

$$Det = 1 - \bar{g}_R \cos\theta + [\bar{g}_D e^{\beta_D (y-y_D)} - \bar{g}_V \beta_V e^{-\beta_V (y-y_V)}]\sin\theta + [\bar{g}_D \beta_D e^{\beta_D (y-y_D)} + \bar{g}_V \beta_V e^{-\beta_V (y-y_V)}]\cos^2\theta$$

The multipliers are roots of the characteristic equation:

$$\mu^2 - Tr\ \mu + Det = 0. \tag{S3}$$

The criterion of fixed point stability requires that both multipliers are located inside the unit circle. A remarkable finding is that for all parameter sets (corresponding to different neuron types and different parts of growing axon), which have been identified by the optimization procedure, the fixed points $(\bar{y}, 0)$ is stable and the fixed point $(\bar{y}, \pi)$ is unstable. It means that the growing axon tends to grow monotonically along the longitudinal axis and the axon asymptotically approaches the dorso-ventral position $\bar{y}$.

Application of this analysis to a specific example of model axon growth. This section illustrates the analytical findings and shows the influence of the random variable on the shape of axon growth. In reality, other factors influence the tendency of axons towards monotonic longitudinal growth near a particular dorso-ventral position. To explore this we again consider the case of ascending axon growth in tadpole aIN neurons. Details of the tadpole CNS, its spinal cord neurons, and the specific adjustments to the equations that must be made to model axon growth in the tadpole were described in the main text above. Values of the environmental parameters are:

$$\beta_D = \ln(10)/30,\ y_D = 145,\ \beta_V = \ln(10)/30,\ y_V = 5.$$

Values of parameters specific for neurons of aIN type are:

$\bar{g}_R = 0.054,\ \bar{g}_D = 0.038,\ \bar{g}_V = 0.13.$

These parameter values have been found by using the stochastic optimization procedure. Substitution of these parameter values to formulas (S2) and (S3) gives the following results:

$\bar{y} = 83.2.$

For the fixed point $(\bar{y}, 0)$ the roots of the characteristic equation are $\mu_1 = 0.99,\ \ \mu_2 = 0.95.$ This means that the fixed point is stable. For the fixed point $(\bar{y}, \pi)$ the roots are the complex conjugate $\mu_{1,2} = 0.97 \pm 0.33i$ and these roots are outside of the unit circle: $|\mu_{1,2}| = 1.02,$ therefore the fixed point is unstable.

Figure S2B shows critical boundaries in the plane of two parameters $(\bar{g}_V, \bar{g}_D)$ for three values of the polarity $\bar{g}_R$.. The black line corresponds to the value $\bar{g}_R = 0.007$ and in the region under the curve (bottom left corner) the fixed point $(\bar{y}, 0)$ is stable. The red line is the critical boundary for $\bar{g}_R = 0.02$ and in the region under this red line the fixed point $(\bar{y}, 0)$ is stable. Respectively, the blue line is the critical boundary for $\bar{g}_R = 0.05$ and in the region under this blue curve the fixed $(\bar{y}, 0)$ is stable. Of course, in each of these three cases the complimentary region shows the instability. Figure S2B clearly shows that the tendency for longitudinal growth is highly stable except towards very low sensitivities for the rostral polarity.
