# Supplementary material for: A Developmental Approach to Predicting Neuronal Connectivity from Small Biological Datasets: A Gradient-Based Neuron Growth Model
Source: PLoS One. 2014 Feb 21;9(2):e89461. doi: 10.1371/journal.pone.0089461 (PMC3931784; doi:10.1371/journal.pone.0089461)
Supplement: Supporting Information S4 — Soma distribution. Method for generating a rostro-caudal distribution of soma positions. (PDF) [file pone.0089461.s005.pdf]

Supporting Information S4. Soma distribution

***Distribution of rostro-caudal soma positions*.** In the region of the tadpole hindbrain and rostral spinal cord considered here as a growth field, there are N neuron somata on one side of the body (*N*=692) of seven types (*j*= 1,2,…,7). The following algorithm provides allocations of their somata (rostro-caudal coordinates) along the growth field starting from position 500 µm till position 2000 µm (Figure shows this for cIN and aIN neuron types). Somata are allocated in discrete positions with step Δ $(here: \Delta = 1.5\ \mu$ m). The total length of 1500 µm is divided to 15 equal bins so the length of each bin b_i , $(i = 1,2,...,15)$ is 100 µm. The number of somata of each type to be allocated to each bin Q_i^j , $(i = 1,2,...,15;\ j = 1,2,...,7$) is based on current anatomical information. The total number of somata $N = \sum_{i=1,15;\, j=1,7} Q_i^j$.

First, by a random permutation of seven numbers, we prescribe an order of neuron-type soma allocation: $[c_1, c_2, c_3, c_4, c_5, c_6, c_7]$, c_k is the number between 1 and 7 indicating the neuron type. This random permutation means that repeating the algorithm of soma allocation results in different soma positions (in fact the number of possible distributions is limited by 7! =5040).

The following procedure is repeated for each rostro-caudal bin i, $(i = 1,2,...,15)$:

1) Somata of type c_1 are evenly distributed along the length of the i th bin and the total number of allocated somatas is $Q_i^{c_1}$.
2) Somata of type c_2 are evenly distributed along the bin. If a position is already occupied by a soma of type c_1 then the immediate right and left positions are considered, and if both of these are occupied then right and left positions on the distance 2Δ are considered, etc. This procedure always converges because the number of positions inside the bin is larger than the total number of somata of all types which should be allocated inside the bin. The total number of allocated somata on this step of the algorithm is $Q_i^{c_2}$.
3) The same procedure is repeated sequentially for somata of other neuron types : $c_3,...,c_7$.

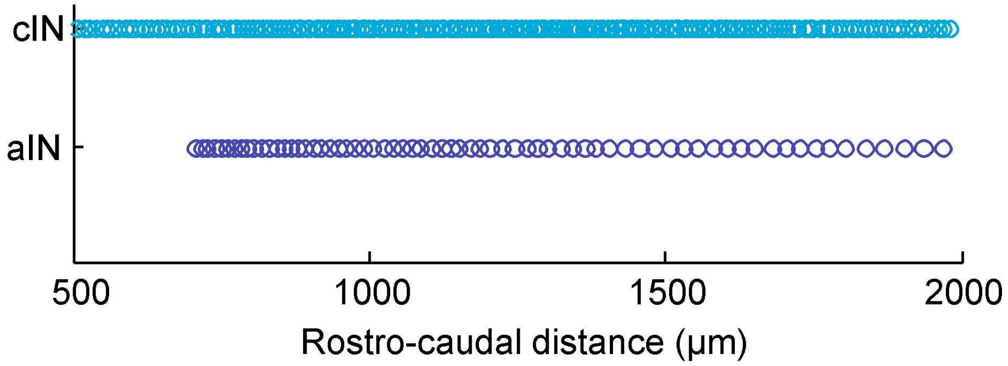

Figure. The distribution of rostro-caudal positions for all 192 cIN neurons and all 63 aIN neurons on one side of the CNS.
